# Supplementary material for: Polyphenols Epigallocatechin Gallate and Resveratrol, and Polyphenol-Functionalized Nanoparticles Prevent Enterovirus Infection through Clustering and Stabilization of the Viruses
Source: Pharmaceutics. 2021 Jul 31;13(8):1182. doi: 10.3390/pharmaceutics13081182 (PMC8398301; doi:10.3390/pharmaceutics13081182)
Supplement: Supplementary file 1 [file pharmaceutics-13-01182-s001.zip › pharmaceutics-1299462-supplementary.pdf]

pharmaceutics

MDPI

Supplementary Materials: Polyphenols Epigallocatechin Gallate and Resveratrol, and Polyphenol-Functionalized Nanoparticles Prevent Enterovirus Infection through Clustering and Stabilization of the Viruses

Dhanik Reshamwala, Sailee Shroff, Olivier Sheik Amamuddy, Valentino Laquintana, Nunzio Denora, Antonella Zacheo [4], Vili Lampinen, Vesa P. Hytonen, Özlem Tastan Bishop, Silke Krol and Varpu Marjomäki

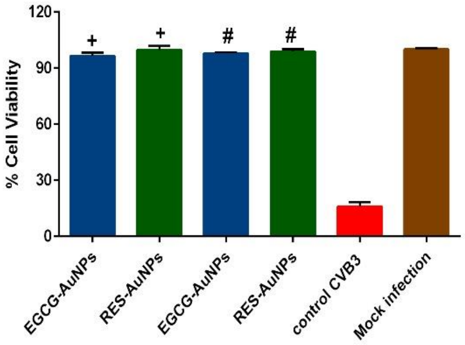

Supplementary figure 1. Testing whether the antiviral efficacy was solely dependent on the ligand bound to the nanoparticles or also from the unbound ligands possibly present in the nanoparticle preparation. In the experiment, AuNP conjugates were ultracentrifuged (10 Psi which corresponds to roughly 50 000 x g, for 30 min) and the pellet was resuspended in ddH2O which was then tested against CVB3 (2 X 10^7 PFU/mL).
+ NPs tested before ultracentrifugation
# NPs tested after ultracentrifugation

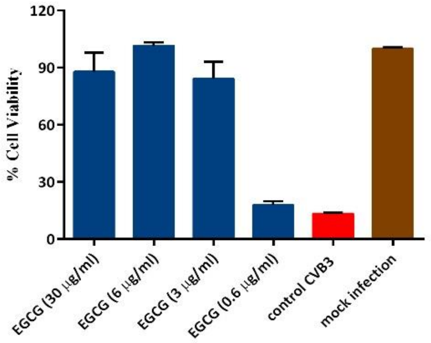

Supplementary figure 2. Testing the antiviral efficacy of EGCG using low viral titer of CVB3 (2 X 10^5 PFU/mL).

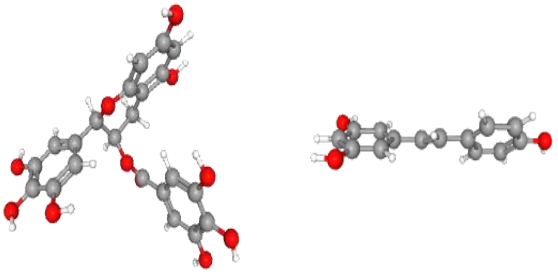

Supplementary figure 3. 3D structure of A) EGCG[1]and B) RES[1].

Supplementary calculation 1: Calculation of number of ligands per nanoparticles.

From the TEM images we assume that the AuNP are spherical. Using the average diameter from the TEM analysis the surface area of the sphere can be calculated by

$$A_{AuNP}=4*\pi*r^2=2*\pi*d^2$$

It is assumed that the 2 aromatic rings (fig. S 3) are oriented parallel to the gold nanoparticle surface (fig. S 3) as we have observed for other aromatic rings in previous experiments [2]. The length and width of the ligand contact area was determined by means of the Avogadro software[1]. The contact area is that of an ellipsoid, hence the area of the ligand is

$$A_{ligand}=\pi*a*b$$

Assuming now the tightest possible ligand packing on the nanoparticle surface, the number N of polyphenols per surface is

$$N= A_{AuNP}/A_{ligand}$$

Supplementary Table 1. Parameters for the calculation of the number of ligands per AuNP

	D* [nm]	A_{AuNP} [nm²]	Ligand a/b# [nm]	A_{ligand} [nm²]	N(ligand/AuNP)
EGCG	131.5 ± 7.1	108 650	0.64/1.14	2.3	47,239
RES	78.6± 2.3	38 817	0.54 /1.2	2.03	19,122

* determined by TEM

# calculated by Avogadro software [1]

Supplementary calculation 2: Calculation of ligand concentration in AuNP stock solution

For the calculation of the ligand concentration bound to AuNP in the stock solution, we calculate first the number of AuNP. For this we calculate first the volume of the spherical AuNP using the radius r, determined in TEM analysis (r=D/2).

$$V_{AuNP}=4/3*\pi*r^3$$

With the nanoparticle volume V_{AuNP} and the density of gold, δ_{Au} we calculated the mass of a single AuNP, m_{AuNP}. The gold content of the stock solution, m_{Au} was measured by ICP-MS. This allows us to calculate the number of AuNP, N_{AuNP} in stock solution by

$$N_{AuNP}= m_{Au}/ m_{AuNP}$$

The calculation reported in table S1 gives us the number of ligand per AuNP, N_{ligand} which allows us to calculate the total number of ligands in the stock solution of gold nanoparticles per L. The concentration of bound ligand to the nanoparticles is calculated by

$$c_{ligand} = N_{ligand} / N_A$$

N_A is the constant of Avogardo (6.02214086 × 10^{23} mol^{-1}); N_{ligand} is the number of ligand per L.

Supplementary Table 2. Parameters for the calculation of the ligand concentration bound to the AuNP in the stock solution.

	D ^ [nm]	V_{AuNP} [nm^3]	V_{NP} [cm^3]	δ_{Au} [g/cm^3]	m_{AuNP} [g]	m_{Au} # [g/L]	N_{AuNP} /L	N_{ligand} /NP	N_{ligand} /L	c_{ligand} [µM]
EGCG	131.5	1,190,627	1.19 * 10^{-15}	19.32	2.30 * 10^{-14}	1.64	7.13 * 10^{13}	47,239	3.37 * 10^{18}	5.61
RES	78.6	254,253	2.54 * 10^{-16}		4.91 * 10^{-15}	5.3	1.08 * 10^{15}	19,122	2.06 * 10^{19}	34.4

^ determined by TEM

determined by ICP-MS

References

[1] Hanwell, M.D.; Curtis, D.E.; Lonie, D.C.; Vandermeersch, T.; Zurek, E.; Hutchison, G.R. Avogadro: An Advanced Semantic Chemical Editor, Visualization, and Analysis Platform. Journal of Cheminformatics **2012**, *4*, 17.

[2] Mandal, S.; Bonifacio, A.; Zanuttin, F.; Sergo, V.; Krol, S. Synthesis and Multidisciplinary Characterization of Polyelectrolyte Multilayer-Coated Nanogold with Improved Stability Toward Aggregation. Colloid Polym. Sci. **2011**, *289*, 269-280.
